# Differences in exam performance between pupils attending selective and non-selective schools mirror the genetic differences between them

**DOI:** 10.1038/s41539-018-0019-8

**Published:** 2018-03-23

**Authors:** Emily Smith-Woolley, Jean-Baptiste Pingault, Saskia Selzam, Kaili Rimfeld, Eva Krapohl, Sophie von Stumm, Kathryn Asbury, Philip S. Dale, Toby Young, Rebecca Allen, Yulia Kovas, Robert Plomin

**Affiliations:** 1 King’s College London, MRC Social, Genetic and Developmental Psychiatry Centre, Institute of Psychiatry, Psychology & Neuroscience, London, SE5 8AF UK; 2 Clinical, Educational & Health Psychology, Division of Psychology & Language Sciences, Faculty of Brain Sciences, University College London, 26 Bedford Way, London, WC1H 0DS UK; 3 London School of Economics and Political Science, Houghton Street, London, WC2A 2AE UK; 4 Department of Education, Psychology in Education Research Centre, University of York, York, YO10 5DD UK; 5 Department of Speech and Hearing Sciences, University of New Mexico, Albuquerque, NM USA; 6 New Schools Network, 3 Albert Embankment, London, SE1 7SP UK; 7 Education Datalab, 1st Floor, 11 Tufton Street, London, SW1P 3QB UK; 8 Laboratory for Cognitive Investigations and Behavioural Genetics, Tomsk State University, Lenin Avenue, 36, Tomsk Oblast, Tomsk, 634050 Russia; 9 Department of Psychology, Goldsmiths, University of London, 8 Lewisham Way, London, SE14 6NW UK

## Abstract

On average, students attending selective schools outperform their non-selective counterparts in national exams. These differences are often attributed to value added by the school, as well as factors schools use to select pupils, including ability, achievement and, in cases where schools charge tuition fees or are located in affluent areas, socioeconomic status. However, the possible role of DNA differences between students of different schools types has not yet been considered. We used a UK-representative sample of 4814 genotyped students to investigate exam performance at age 16 and genetic differences between students in three school types: state-funded, non-selective schools (‘non-selective’), state-funded, selective schools (‘grammar’) and private schools, which are selective (‘private’). We created a genome-wide polygenic score (GPS) derived from a genome-wide association study of years of education (*EduYears*). We found substantial mean genetic differences between students of different school types: students in non-selective schools had lower *EduYears* GPS compared to those in grammar (*d* = 0.41) and private schools (*d* = 0.37). Three times as many students in the top *EduYears* GPS decile went to a selective school compared to the bottom decile. These results were mirrored in the exam differences between school types. However, once we controlled for factors involved in pupil selection, there were no significant genetic differences between school types, and the variance in exam scores at age 16 explained by school type dropped from 7% to <1%. These results show that genetic and exam differences between school types are primarily due to the heritable characteristics involved in pupil admission.

## Genetic differences between selective and non-selective schools

Students attending selective schools have, on average, more genetic variants associated with educational attainment compared to students attending non-selective schools. A team led by Professor Robert Plomin at King’s College London found that these genetic differences between school types were also mirrored in examination differences. Students attending selective schools were performing a grade higher than their non-selective schooled peers. However, once the researchers statistically accounted for student-level factors, including family socioeconomic status, prior ability and prior achievement, there were no significant genetic differences between students in selective and non-selective schools, and only small examination score differences. This research shows that genetic and exam score differences between selective and non-selective schools are primarily due to the genetically influenced characteristics involved in student admission.

## Introduction

Achievement at the end of full-time compulsory education represents a major tipping point in life, opening up avenues for higher education, including university and beyond. Therefore, understanding the potential predictors of academic achievement at this juncture is of great importance. One such predictor that has been hotly debated is school type. In England, when students transition from primary to secondary school at age 11, they have the option of attending one of three school types. Ninety-three percent of children attend state-funded schools, the majority of which are non-selective^1^ (state non-selective). A small proportion of state-funded schools (163 schools out of 3113 schools in England) are academically selective ‘grammar’ schools. These schools select their intake based on achievement and ability, assessed by an entrance exam. The remainder of students (approximately 7%), are private educated. As well as being fee-paying, private schools are often also academically selective. These school types are assumed to set children on different trajectories, with research linking selective schools (grammar and private schools) to later success, including higher levels of academic achievement, acceptance at university, and even higher earning potential compared to pupils educated in non-selective schools.^2–4^

However, by design, selective schools are able to choose their student intake based on certain pupil characteristics. This can include selection on ability or achievement on an entrance test; both of which have been shown to correlate positively with life outcomes, including later academic achievement.^5,6^ Furthermore, by virtue of being fee-paying, entrance into private schools is usually dependent on whether the family can afford it (their socioeconomic status (SES)), which also correlates with future outcomes.^7–10^ Even for state schools, family SES may play a role in what school type a student attends, with grammar schools typically located in more affluent areas and attracting higher SES students on average.^11^ It is, therefore, possible that improved outcomes for pupils in selective schools do not necessarily reflect a higher quality of education, but may simply be the consequence of selection—either active, as in the case of ability or achievement, or passive, as in the case of family SES.

Given the considerable fees charged by private schools, in addition to the potential stress of selective school entrance exams, why do families choose these schools? Among the many possible reasons is superior academic achievement. The finding that pupils at selective schools outperform their non-selective school counterparts in exams has been frequently reported.^2–4,12,13^ At age 16, students in the UK typically take the General Certificate of Secondary Education (GCSE) exams. The UK Department for Education shows that 99% of grammar school students obtain top GCSE grades (A*–C grade) in English and mathematics, compared to 64% for all state-funded mainstream school students.^14^ However, academic achievement at age 16 is positively correlated with the factors involved in pupil selection, such as prior achievement, ability and SES.^6,15^ Therefore, this raises the question—are selective schools adding anything over and above these factors in the prediction of academic achievement?

Several studies have attempted to elucidate the effect of school type on achievement over and above factors on which schools can select (for example,^13,16^ for a review, see Coe et al.).^3^ However, many of these have not been published in peer-reviewed journals—for example^2,3,17,18^—and we are not aware of a recent peer-reviewed study looking at all three school types: state non-selective, grammar and private schools in the UK. However, the non-peer-reviewed reports support the conclusion that there are only small academic advantages to attending a selective school, after student factors such as achievement, ability and family SES have been taken into account.

Traditionally, the relationship between the factors involved in school admission and later achievement have been thought to operate environmentally. For example, parents with higher SES may invest more time in their children’s education^19^ and can afford more resources (e.g., more books or private tuition), which in turn may lead to better opportunities and improved achievement. However, a less frequently investigated factor influencing both selection factors, as well as achievement, is genetics. In the example above, parents with higher SES are not only passing on educationally relevant environments, but they are also passing on educationally relevant genes, a concept referred to as gene-environment correlation (rGE).

A vast literature from quantitative genetics has shown that genetic factors explain a substantial amount of variance in selection factors, including ability and achievement.^20–23^ Heritability estimates of general cognitive ability (*g*) from twin studies range from around 30% in childhood, to 40–50% in adolescence and approximately 60% in adulthood.^21^ Twin studies also show that much of the relationship between selection factors, such as *g*, and later achievement, are substantially influenced by genetics.^22–26^ Because twins typically grow up in the same family, the aetiology of traits such as family SES, which do not vary between twins, cannot be estimated in this way. However, heritability can be estimated by genome-wide complex trait analysis (GCTA),^27,28^ which uses DNA from unrelated individuals to estimate the proportion of phenotypic variance explained by hundreds of thousands of single-nucleotide polymorphisms (SNPs) genotyped on DNA arrays. This method has also shown that genetics accounts for a significant amount of individual differences in family SES,^29,30^ as well as *g* and achievement.^31–33^

School type, like SES, does not tend to vary within twin pairs. However, because GCTA requires large sample sizes, it has so far not been possible to look at the genetic differences between students of different school types. However, powerful genome-wide association (GWA) studies of behavioural traits, which test associations between specific SNPs and traits are starting to make this possible. Although individually these SNPs, identified through GWA studies, are of small effect, by summing their effects together it is possible to create a genetic score for each individual in an independent sample, which explains a substantial proportion of the genetic variation.^34–36^ These scores, dubbed ‘genome-wide polygenic scores’ (GPS) are a game-changer for genetic research and have already proved insightful within the area of educational achievement. For example, a recent study^37^ using a GPS derived from a 2016 GWA study of years of education (*EduYears*)^38,39^ has shown educational achievement scores at age 16 differ as a function of GPS. There was approximately one standard deviation difference between those in the highest GPS septile and those in the lowest; representing almost a whole school grade difference. Furthermore, while 65% of students in the highest GPS septile went on to university, only 37% in the lowest septile progressed to university-level education.

For the first time, we assess differences in a polygenic score for years of education (*EduYears)* between students from three school types: non-selective, grammar and private schools. We predict that selection involving heritable traits such as achievement, ability and family SES will be reflected in the genetic differences between students of different school types. Furthermore, in line with previous literature, we expect that selection will also create large achievement differences between students attending the three school types, which will reduce substantially once controlling for the selection factors.

## Results

### Polygenic score differences between school types

**Fig. 1:**
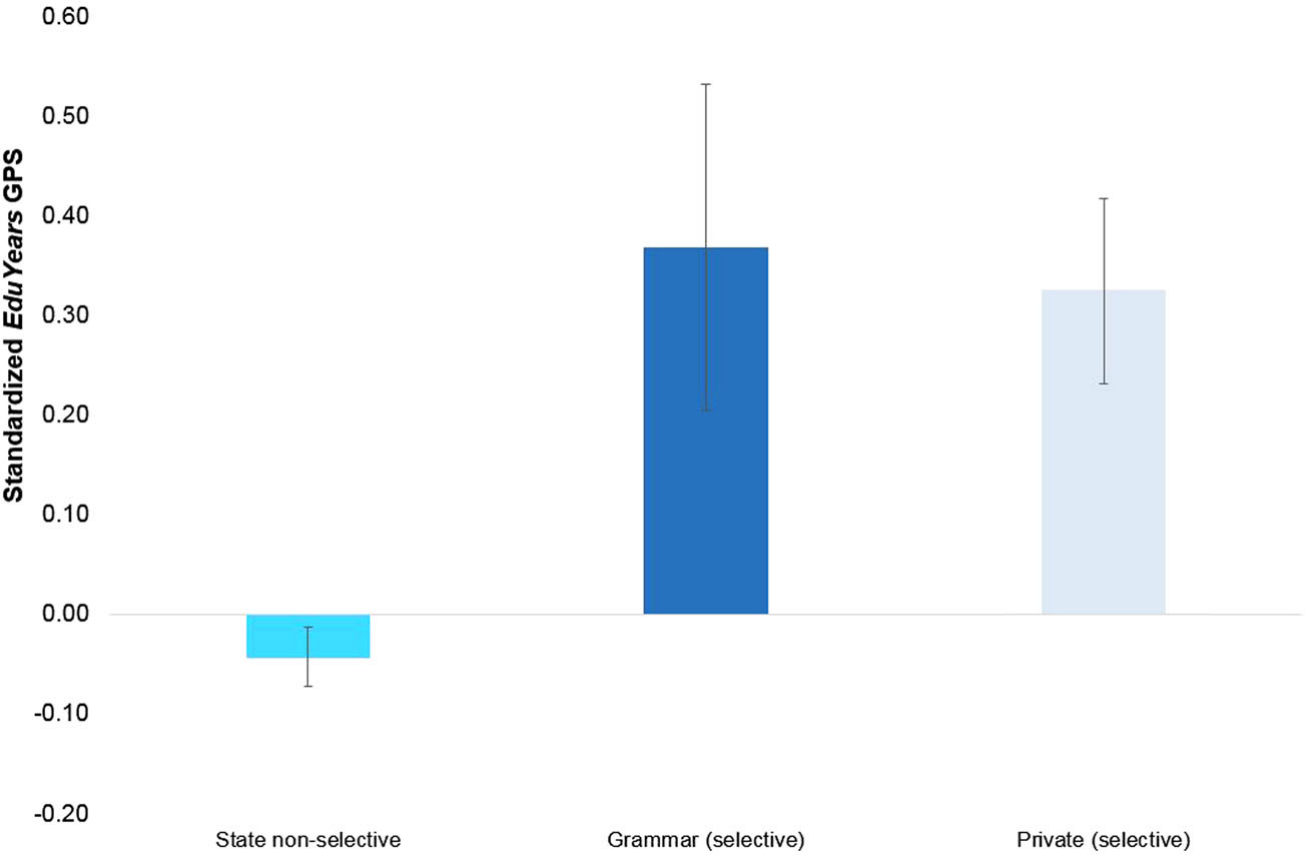
*EduYears* GPS plotted means (and 95% confidence intervals) between state non-selective, grammar and private school students. *Note:* There were significant *EduYears* GPS mean differences between state non-selective school students and both grammar (*t* = 4.869, *p* < 0.001; *d* = 0.413) and private school students (*t* = 7.170, *p* < 0.001; *d* = 0.372). There was not a significant difference between grammar and private school students (*t* = 0.436, *p* = 0.659)

Students attending different school types (state non-selective, grammar and private schools) differed genetically, as shown by their mean *EduYears* GPS (see Fig. 1, analysis of variance (ANOVA) details in Table S1). Non-selective state school students had significantly lower *EduYears* GPS scores compared to grammar school students (*t* = 4.87, *p* < 0.001) and private school students (*t* = 7.17, *p* < 0.001). These differences translate to more than a third of a standard deviation difference (*d* = 0.41 and 0.37, respectively). There were no significant mean differences in *EduYears* GPS scores between grammar and private school students (*t = *0.44, *p* = 0.66). There were also no significant mean differences between state non-selective schools in varying selectivity areas (see Table S2 and Supplementary Fig. S1).

### Associations between EduYears GPS and selection factors

*EduYears* GPS was positively correlated with each of the selection factors (see Supplementary Table S3), explaining 2.1% of the variance in ability, 5.2% in achievement and 6.6% in family SES. *EduYears* GPS was also positively correlated with GCSE, explaining 7.6% of the variance in GCSE scores, similar to previous analysis of these data.^37^ Because selective schools actively select for achievement and ability and passively select for SES, all of which correlate with *EduYears* GPS, we tested whether mean differences in EduYears GPS remained once controlling for these factors.

We found that, after accounting for the variance explained by heritable selection factors, there were no significant *EduYears* GPS differences between students of the three school types: state non-selective, grammar and private (see Supplementary Fig. S2 and Supplementary Table S4). Similar results also emerged when we looked at differences between state non-selective schools in varying selectivity areas (see Supplementary Table S5 and Supplementary Fig. S3), showing small differences in *EduYears* between school types.

### GCSE differences

**Fig. 2:**
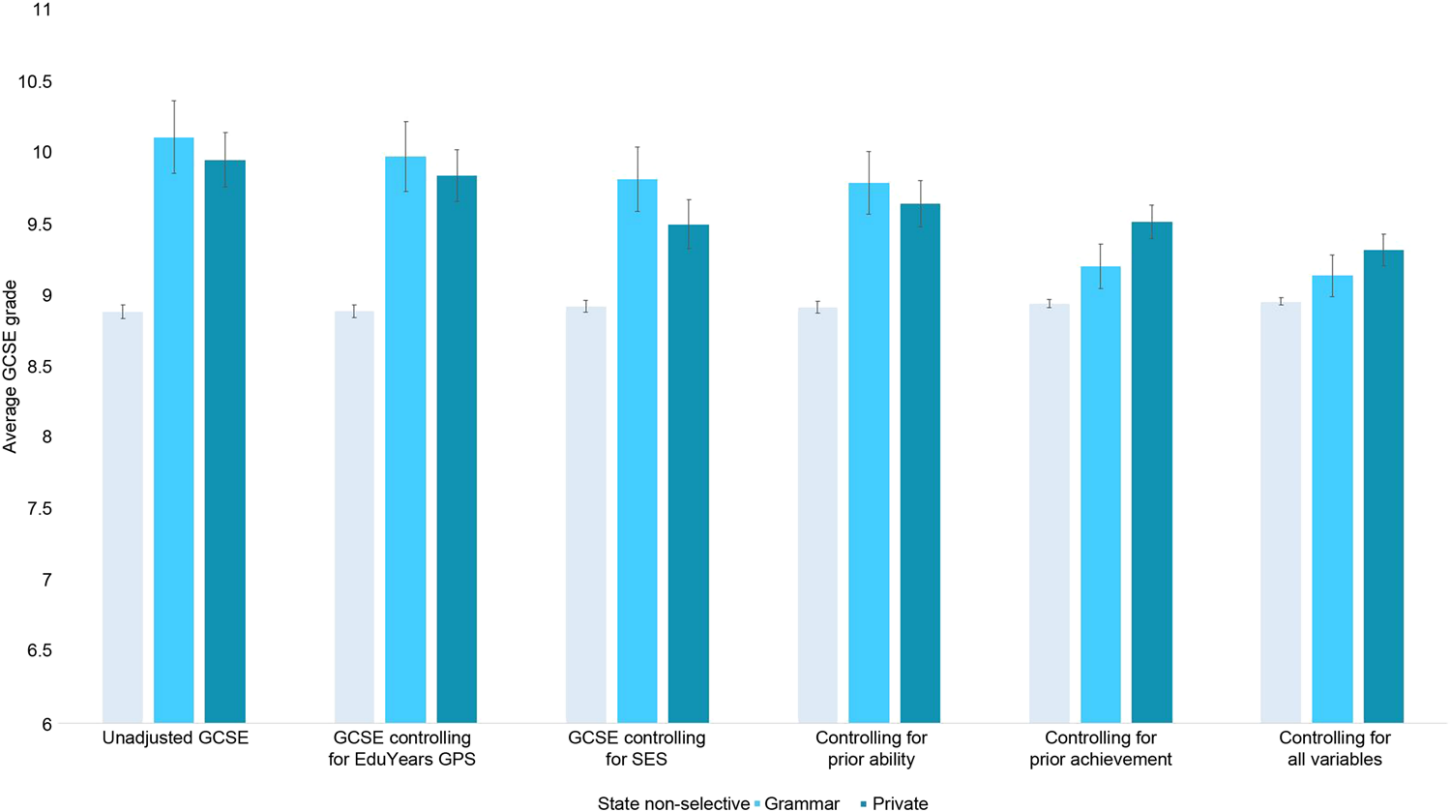
Plotted means (and 95% confidence intervals) for unadjusted GCSE, GCSE controlling for GPS, GCSE controlling for SES, GCSE controlling for prior ability, GCSE controlling for prior achievement and GCSE controlling for all variables between three school types: state non-selective, grammar and private. *Note:* Details can be found in Supplementary Table S6

Supplementary Table S6 and Fig. 2 show unadjusted average GCSE grades for state non-selective, grammar and private school students, as well as average GCSE score adjusting separately for *EduYears* GPS, family SES, prior ability and prior achievement, and for all variables together. Unadjusted GCSEs between school types mirrored unadjusted *EduYears* GPS results, with large differences between non-selective and selective schools (see ‘Unadjusted GCSE’ in Fig. 2, details in Supplementary Table S6). Indeed, the mean GCSE score of students attending state non-selective schools was approximately 1 SD below the mean GCSE score of those attending grammar schools (*d* = 1.05, 95% CIs = 0.83–1.28) and private school students (*d* = 0.92, 95% CIs = 0.75–1.09). This translates to around a whole grade difference between average GCSE scores for state non-selective school students and selective school students. There was no difference between grammar and private school students’ average GCSE score (*t = *1.00, *p* = 0.32). There were also no significant differences between non-selective schools in areas that varied in the selectivity of their schools (see Supplementary Table S7 and Supplementary Fig. S4).

### Controlling for selection factors

Controlling for *EduYears* GPS had a small effect on average GCSE grades, with the GCSE variance explained by school type dropping slightly from *R*^*2*^ = 0.07 to 0.06, see Fig. 2, details in Supplementary Table S6). This relatively small effect is to be expected given that *EduYears* GPS accounts for only 8% of the variance in GCSE (see Supplementary Table S3). Controlling for family SES and prior ability had a slightly larger effect on GCSE, in line with the GCSE variance they account for (*R²* = 24% and 27%, respectively). Out of all of the selection factors, prior achievement had the biggest impact on GCSE grades between school type, with average GCSE for grammar schools falling from 10.12 (grade A) to 9.21 (grade B). After controlling for prior achievement, the variance in GCSE explained by school type dropped from 7.1 to 1.3%.

Controlling for all of the selection factors and *EduYears* GPS together saw a further reduction in average GCSE between school types, with average GCSE score for grammar (*M* = 9.14; *t* = 2.35, *p* < 0.019) and private (*M* = 9.32, *t* = 6.16, *p* < 0.001) similar to that of state non-selective school students’ average grade (*M* = 8.96). Although these mean differences between school types remained significant, they were greatly reduced. Standardised betas indicated that attending a grammar school compared to a non-selective state school was associated with an increase of just 0.03 of a standard deviation in GCSE, and for private schools, the increase was 0.07. In addition, no significant differences emerged between non-selective schools in varying selectivity areas (see Supplementary Table S7 and Supplementary Fig. S4).

One of our main findings was that after accounting for the variance explained by the selection factors and *EduYears* GPS, the variance in GCSE explained by school type dropped from 7.1% to only 0.5% (see Supplementary Table S6 for regression results).

## Discussion

We report genetic mean differences between students attending three different types of school: state non-selective, grammar and private schools. We find that, on average, students in state non-selective schools have lower polygenic scores for years of education (*EduYears*) compared to their peers in selective schools. Furthermore, following the same pattern of results as *EduYears*, there are also substantial mean differences in GCSE performance between pupils in selective and non-selective school types. However, almost all of these differences are explained by heritable, individual-level factors, which schools actively or passively use in the pupil selection process.

Although finding genetic differences between state non-selective, grammar and private school students may initially seem surprising, when we consider the heritable traits that selection is based on, this difference is less unexpected. Put another way, students with higher polygenic score for years of education have, on average, higher cognitive ability, better grades and come from families with higher SES, and these students are subsequently more likely to be accepted into selective schools. This results in a system in which children are intentionally phenotypically selected, but unintentionally genetically selected.

However, despite finding mean genetic differences between students of different school types, it should be noted that the majority of the variation in *EduYears* GPS occurs within the school type, not between the school types. For example, a Cohen’s *d* of 0.41, (the difference between mean *EduYears* scores for state non-selective school students and grammar school students), which is classed as a small-medium effect size, translates to an overlap of approximately 83% between the two distributions.^40^

Nevertheless, finding an association between genotype and school type suggests that genetic factors are contributing to variation in educational environments, a concept known as gene-environment correlation (rGE). This occurs when individuals select, modify and ‘inherit’ their environment, in part based on their genotype.^20,41^ Putting our research within the context of rGE, we suggest that in addition to students being selected into schools based on their genetically influenced traits (evocative rGE), children themselves also actively select educational environments that correlate with their genotype (active rGE). In the case of high achieving students, these environments might be challenging or competitive academic institutions, which grammar and private schools are often reputed to be. Finally, because we know that the factors used in school selection are substantially heritable, it is likely that academically gifted children will come from academically gifted parents. These parents not only provide the genes but also the environments to help them progress academically.

As well as having a higher average *EduYears* polygenic score, students attending selective schools also achieve better GCSE results on average.^2,3,12–14,17^ There has been some debate in the literature as to the size of this achievement gap, with studies accounting for different background characteristics in their analysis. We find that almost all of the selective school advantage in GCSE can be explained by family SES, achievement, ability and *EduYears GPS*. After controlling for these factors, going to a grammar vs. a state non-selective school is associated with a mean GCSE grade increase of just 0.026 of a standard deviation and for private schools, 0.070 of a standard deviation. Furthermore, the variance in GCSE that school type explains falls from 7% to <1%.

Controlling for *EduYears* alone had a fairly small effect on average GCSE grades between school types. However, this is to be expected considering that *EduYears* GPS currently predicts approximately 8% of the variance in GCSE—15% of the heritability estimated by the twin design^22^ and approximately one-third of the heritable variance from SNP-based studies of GCSE at age 16.^30^ The predictive nature of *EduYears* is likely to increase with more powerful GWA studies. For example, there was a threefold increase in prediction of educational achievement at age 16 from the 2016 *EduYears* GPS (based on a GWA study with *N* = 293,723) as compared to the 2013 *EduYears* GPS (*N* = 126,559).^37^

Although there were only small mean differences between school types once selection factors and *EduYears* were controlled for, this does not mean that other factors are not important for achievement at age 16. Altogether, these factors do not predict all of the variance in GCSE (*R*² = 0.69). As shown previously, achievement is the result of many genetically influenced traits, including behaviour, personality, home environment and health.^22^ Furthermore, by finding a small effect of school type, we are not saying schools are unimportant, or that teaching does not work. Without schools, it is hard to imagine a successful education system that allows children to reach their academic potential. However, while schools themselves are important for academic achievement, the type of school appears less so. Educational achievement is not necessarily the only reason parents opt to send their children to selective schools. A recent report on private schools found that these students earned about £200,000 more in their early career (between ages 26 and 42) as compared to state school students.^2^ However, this report did not distinguish between non-selective and selective state schools. More research is needed to see whether differences in university attendance, career choice and earnings are still predicted by school type once individual student factors have been accounted for. In addition to differences in university and career outcomes, it would also be of interest to identify potential differences between school types in terms of non-cognitive traits as outcomes, with one survey finding 66% of parents believing that private schools ‘instil a sense of confidence in pupils’.^2^

There are several limitations to our study. First, we recognise that there is considerable variation in schools within our three school types—within each of the school types, there will be examples of exceptional and under-performing schools. In particular, there is more variance in the state non-selective schools category as it includes most of the schools. It also includes a wide variety of other categories, such as schools that are allowed to select for religion and schools that are allowed to select up to 10% of their pupils for talent in specialist subjects, such as sport, performing or visual arts, and languages. These schools are not allowed to select directly on academic grounds. However, there is some evidence that they do in fact select more able students.^42^ Nonetheless, accounting for prior achievement and ability at age 11, before most children enter secondary school, adjusts for this.

Another limitation of the present study is access to school type. Grammar and private schools are not evenly distributed around the country. Therefore, in local authority areas where there are no selective schools, the average GCSE grade of pupils in non-selective schools may be higher and in areas where there are a greater number of selective schools, the average GCSE grade of non-selective schools may be lower. Because there are far fewer selective schools, this geographical effect may potentially inflate the average non-selective school GCSE grade. To see whether this had an impact on GCSE differences, we split the non-selective school group into three further groups: non-selective schools in selective areas, partially selective areas and non-selective areas. Once we controlled for all of the selection factors, we found that there were no differences between non-selective schools in areas of varying selectivity (see Supplementary Table S7 and Supplementary Fig. S4).

A final limitation to note is that the GCSE variable we used in the analysis is a composite of only the three core subjects taken at age 16—English, science and mathematics. For other subjects, such as languages, art and social sciences, school type may have a greater influence. However, because different school types prioritise different subjects,^43^ it is difficult to untangle the effect of school type on optional rather than core subjects, although this would be a useful direction for future research.

In the current study, we find genetic differences between students attending three school types: state non-selective schools, grammar schools and private schools. We find that selective school students have higher polygenic scores for years of education on average compared to students attending non-selective schools. Furthermore, we find substantial mean differences in GCSE between school types. However, once student and family factors have been accounted for, as well as *EduYears* GPS, the type of school that a child attends explains less than one percent of the individual differences in educational achievement (GCSE mean grade) at age 16.

## Methods

### Sample

This study included unrelated individuals from the Twins Early Development Study (TEDS). TEDS is a large, representative sample of 16,000 twin pairs born in England and Wales between 1994–1996 and followed from birth to the present day.^44^ Ethical approval for this study was received from King’s College London Ethics Committee. Although there has been some attrition throughout the years, approximately 10,000 twin pairs are still actively involved in the study and provide rich behavioural and cognitive data. Importantly, TEDS was and still is a representative sample of England and Wales, as described in detail elsewhere.^44,45]^ In the present study, we included 4814 unrelated individuals (one twin randomly in a pair) who had data present for three key variables: genotype data, educational achievement at age 16 and school type data. This sample included 2597 females (54%) and 2217 males (46%). Of this sample, 2533 individuals also had data present for the selection factors: ability, achievement and SES, which included 1427 females (56.3%) and 1106 males (43.7%). For a breakdown of sample sizes by school type, see Supplementary Table S8. Written informed consent was given for all participants involved for each wave of data collection.

### Genotyping

For information on how the sample were genotyped and the quality control process, please see Supplementary Methods S1.

### Measures

#### School type

When TEDS twins were 18, they received a questionnaire that included a series of questions asking what type of school they attended when they took exams at age 16—the GCSEs. Respondents were asked to indicate either ‘Yes’ or ‘No’ for different school types. We classified all respondents who reported attending either a state non-selective school as ‘State non-selective’, all those who indicated that they went to a grammar school as ‘Grammar’ and all those indicating that they went to a private school as ‘Private’. In addition to TEDS data, we also accessed school type information through the National Pupil Database (NPD; https://www.gov.uk/government/collections/national-pupil-database). By supplementing TEDS data with that from NPD, our final school type numbers were: state non-selective: *n* = 4263, grammar: *n* = 143, private: *n* = 408. We also further split state non-selective schools into three categories for follow-up analysis: non-selective schools in fully selective areas (*n* = 331), non-selective schools in partially selective areas (*n* = 905) and non-selective schools in non-selective areas (*n* = 3027). For more information on how and why we created these groupings, including accuracy between data sources and selective area groupings, please see Supplementary Methods S2.

#### Educational achievement at age 16

The GCSE is a standardised UK-based examination administered at the end of compulsory education at age 16 (*M = *16.31, SD* = *0.29). Almost all students take the three core subjects: English, mathematics and science. In addition, students are allowed to choose a range of other subjects such as geography, history and art. These subjects were graded from 4 (G, the minimum pass grade) to 11 (A*, the best possible grade). In the current sample, GCSE results were obtained from questionnaires sent via mail, in addition to telephone interviews with twins and their parents. We further supplemented this with data from NPD. Our analyses focused on the three core subjects: English, mathematics and science taken by all students. Students taking science GCSE are either awarded separate GCSEs for physics, chemistry and biology (‘triple science’) or as one course, which is double weighted (‘double science’), therefore, we took a mean grade of the science GCSEs. Because English, mathematics and science grades correlated highly (*r* = 0.70–0.82), we created a GCSE composite. There were 3920 individuals for whom we had both self-reported GCSE and NPD data, this composite correlated at *r = *0.99 between both data sources, which supported the high accuracy of TEDS data.

### Selection factors

#### Socioeconomic status

Family SES was measured by taking the arithmetic mean of five measures: maternal and paternal education (measured on a scale from 1–8, where 1 = no education and 8 = postgraduate qualifications), occupation (indexed by the Standard Occupational Classification (2000) on a scale from 1–9, where 1 = elementary administration and service occupations and 9 = managers, directors and senior officials) and maternal age at birth of first child. All measures were standardised to have a mean of 0 and a SD of 1 and at least three measures were required to calculate the arithmetic mean.

#### Achievement tests at age 11

We did not have access to selective school entrance exams, however, before children transition to secondary school, they are usually required to take exams, which include English and mathematics tests. In our sample these tests comprise two English tests (reading and writing) and three maths tests (calculator and non-calculator test as well as a mental arithmetic test). Due to the high correlation between maths and English scores (*r = *0.67), we created a composite of these test scores requiring both to be present.

#### Ability (general cognitive ability, *g*)

To measure general cognitive ability, participants were asked to complete an online battery of cognitive tests administered as part of TEDS testing at age 11. These tests included verbal and non-verbal abilities (*M = *11.2, SD = 0.69). A mean score was derived from four tests, two verbal tests (the Wechsler Intelligence Scale for Children (WISC) Vocabulary Multiple-Choice and the WISC General Knowledge test)^46^ and two non-verbal tests (Raven’s Progressive Matrices^47^ and the WISC Picture Completion task).^48^

### Data availability

For information on data availability, please see the Twins Early Development Study data access policy. This can be found at: http://www.teds.ac.uk/research/collaborators-and-data/teds-data-access-policy.

### Analyses

#### Genome-wide polygenic scores

We calculated polygenic scores that were based on the summary statistics of the largest GWA study for years of education (*N* = 293,723 individuals).^39^ A GPS is calculated by using information from GWA study summary statistics about the strength of association between a genetic variant and a trait, to score individuals’ genotypes in independent samples. For each genotype in the independent sample, all trait-associated alleles are counted and multiplied by their effect size (i.e., their strength of association with a trait as reported in GWA summary statistics). The sum of these weighted and counted alleles forms a polygenic score for each individual. We used the software PRSice to create individual GPS. Those SNPs that passed quality control were clumped for linkage disequilibrium by applying an *R*^2^ = 0.1 cutoff within a 250-kb window. It is possible to calculate various GPS based on different GWA study significance thresholds for genetic variants, with less stringent *p*-value thresholds resulting in GPS that include more SNPs. Here, we calculated GPS for seven *p*-value thresholds (0.001, 0.05, 0.1, 0.2, 0.3, 0.4, 0.5). We report analyses for the *p*-value threshold of 0.05 in the main text; however, the analyses for the other *p*-value thesholds are reported in Supplementary Fig. S5. We regressed all GPS on the first ten principal components and used these standardised residuals in our analyses to account for population stratification.

#### Mean differences

To estimate differences between the three school types: state non-selective, grammar and private schools, we used a one-way ANOVA with planned contrasts. In addition to the three-level school type analysis, we also conducted follow-up analysis looking at differences between state non-selective schools in areas with and without grammar schools: non-selective schools in fully selective areas, non-selective schools in partially selective areas and non-selective schools in non-selective areas. As the sample sizes varied between groups, we used adjusted Cohen’s *d* to estimate effect size. This test adjusts the calculation of the pooled standard deviation with weights for the sample sizes.

To test the effect of school type after controlling for selection factors (SES, prior achievement and prior ability) and *EduYears* GPS, we conducted hierarchical linear regression with dummy coding. See Supplementary Methods S3 for further information on analysis.

All methods were performed in accordance with relevant regulations and guidelines.

## Electronic supplementary material

Supplementary Materials

## Electronic supplementary material

**Supplementary information** accompanies the paper on the *npj Science of Learning* website (10.1038/s41539-018-0019-8).
