# Supplementary material for: Differences in exam performance between pupils attending selective and non-selective schools mirror the genetic differences between them
Source: NPJ Sci Learn. 2018 Mar 23;3:3. doi: 10.1038/s41539-018-0019-8 (PMC6220309; doi:10.1038/s41539-018-0019-8)

Supplementary Online Material

Differences in exam performance between pupils attending selective and non-selective schools mirror the genetic differences between them

Smith-Woolley, E., Pingault, J B., Selzam, S., Rimfeld, K., Krapohl, E., von Stumm, S., Asbury, K., Dale, P. S., Young, T., Allen, R., Leunig, T., Kovas, Y., & Plomin, R.

Supplementary methods: **Page**

Methods S1 – Details on genotyping 2
Methods S2 – Creating the school type variables 4
Methods S3 – Hierarchical linear regression to calculate adjusted means for school type 8

Supplementary Tables:

Table S1 – Analysis of variance (ANOVA) and planned contrasts for *EduYears* GPS between students of three school types: state non-selective, grammar and private schools 9
Table S2 – Analysis of variance (ANOVA) and planned contrasts for *EduYears* GPS between students of five school types: non-selective schools in wholly selective areas, non-selective schools in partially selective areas, non-selective schools in non-selective areas, grammar schools and private schools 10
Table S3 – Correlation matrix 11
Table S4 – Hierarchical regression analysis of *EduYears* GPS, controlling for selection factors for students of three school types: state non-selective, grammar and private schools 12
Table S5 – Hierarchical regression analysis of EduYears GPS, controlling for selection factors for students of five school types: non-selective schools in wholly selective areas, non-selective schools in partially selective areas, non-selective schools in non-selective areas, grammar schools and private schools 13
Table S6 – Regression analysis of predictors of mean GCSE for three school types: state non-selective, grammar and private schools 14
Table S7 – Regression analysis of predictors of mean GCSE for three school types: non-selective schools in wholly selective areas, non-selective schools in partially selective areas, non-selective schools in non-selective areas, grammar schools and private schools 16
Table S8 – Descriptive statistics 18

Supplementary Figures:

Figure S1 – *EduYears* GPS plotted means (and 95% confidence intervals) for students of five school types: non-selective schools in wholly selective areas, non-selective schools in partially selective areas, non-selective schools in non-selective areas, grammar schools and private schools 19
Figure S2 – *EduYears* GPS plotted means (and 95% confidence intervals) controlling for selection factors between students of 3 school types: non-selective state, grammar and private 20
Figure S3 – EduYears GPS plotted means (and standard errors) controlling for selection factors between 5 school types: non-selective schools in wholly selective areas, non-selective schools in partially selective areas, non-selective schools in non-selective areas, grammar schools and private school 21
Figure S4 – The plotted means (and 95% confidence intervals) for unadjusted GCSE, GCSE controlling for *EduYears* GPS, GCSE controlling for SES, GCSE controlling for prior ability, GCSE controlling for prior achievement and GCSE controlling for all variables between 5 school types: non-selective schools in wholly selective areas, non-selective schools in partially selective areas, non-selective schools in non-selective areas, grammar schools and private school 22
Figure S5 – Mean *EduYears* GPS (and 95% confidence intervals) between state non-selective, grammar and private school for several EduYears GPS p-value cut-off 23

Methods S1 – Details on genotyping:

DNA for 4,649 individuals was extracted from saliva and buccal cheek swab samples and hybridized to HumanOmniExpressExome-8v1.2 genotyping arrays at the Institute of Psychiatry, Psychology and Neuroscience Genomics & Biomarker Core Facility. The raw image data from the array were normalised, pre-processed, and filtered in GenomeStudio according to Illumina Exome Chip SOP v1.4. (http://confluence.brc.iop.kcl.ac.uk:8090/display/PUB/Production+Version%3A+Illumina+Exome+Chip+SOP+v1.4). In addition, prior to genotype calling, 869 multi-mapping SNPs and 353 samples with call rate <.95 were removed.

DNA from 3,665 samples was extracted from buccal cheek swabs and genotyped at Affymetrix, Santa Clara, California, USA. Samples were successfully hybridized to AffymetrixGeneChip 6.0 SNP genotyping arrays (http://www.affymetrix.com/support/technical/datasheets/genomewide_snp6_datasheet.pdf) using experimental protocols recommended by the manufacturer (Affymetrix Inc., Santa Clara, CA). The raw image data from the arrays were normalized and pre-processed at the Wellcome Trust Sanger Institute, Hinxton, UK for genotyping as part of the Wellcome Trust Case Control Consortium 2 (https://www.wtccc.org.uk/ccc2/) according to the manufacturer's guidelines (http://www.affymetrix.com/support/downloads/manuals/genomewidesnp6_manual.pdf). Genotypes for the Affymetrix arrays were called using CHIAMO (https://mathgen.stats.ox.ac.uk/genetics_software/chiamo/chiamo.html).

After initial quality control and genotype calling, the same quality control was performed on the samples genotyped on the Illumina and Affymetrix platforms separately using PLINK[1,2], R[3], and vcftools[4].

Samples were removed from subsequent analyses on the basis of call rate (<0.99), suspected non-European ancestry, heterozygosity, array signal intensity, and relatedness (IBD cut-off 0.05). SNPs were excluded if the minor allele frequency was <0.5%, if more than 1% of genotype data were missing, or if the Hardy Weinberg *p*-value was lower than 10^{-5}. Non-autosomal markers and indels were removed. Association between the SNP and the platform, batch, or plate on which samples were genotyped was calculated; SNPs with an effect *p*-value less than 10^{-3} were excluded. A total sample of 6,710 samples, with 3,617 individuals and 600,034 SNPs genotyped on Illumina and 3,093 individuals and 525,859 SNPs genotyped on Affymetrix remained after quality control.

Genotypes from the two platforms were separately imputed using the Haplotype Reference Consortium [5] and Minimac3 1.0.13 [6,7] available on the *Michigan Imputation Server* as reference data. A series of quality checks was performed before merging data from the two platforms' imputation (e.g. platform effects, allele frequencies by imputation quality). For the present analyses we limited our analyses to variants genotyped or imputed at info >.70 on both platforms, allele frequency difference between platforms smaller than 5%, and Hardy Weinberg *p*-value was greater than 10^{-5}. Using these criteria, 7,581,516 genotyped and well-imputed SNPs were retained for the analyses.

We performed principal component analysis on a subset of 42,859 common (MAF>5%) autosomal HapMap3 SNPs [8], after stringent pruning to remove markers in linkage disequilibrium ($r^2 > 0.1$) and excluding high linkage disequilibrium genomic regions so as to ensure that only genome-wide effects were detected.

Of the final sample of successfully genotyped individuals, there were 4,814 people who also had information on school type and exam results at age 16 which were included in the present analysis.

References

1. Chang, C. C. *et al.* Second-generation PLINK: rising to the challenge of larger and richer datasets. *GigaScience* **4,** 7 (2015).
2. Purcell, S. *et al.* PLINK: A Tool Set for Whole-Genome Association and Population-Based Linkage Analyses. *Am. J. Hum. Genet.* **81,** 559–575 (2007).
3. R Core Team. *R: A Language and Environment for Statistical Computing*. (R Foundation for Statistical Computing, 2015).
4. Danecek, P. *et al.* The variant call format and VCFtools. *Bioinformatics* **27,** 2156–2158 (2011).
5. McCarthy, S. *et al.* A reference panel of 64,976 haplotypes for genotype imputation. *bioRxiv* 035170 (2015). doi:10.1101/035170
6. Howie, B., Fuchsberger, C., Stephens, M., Marchini, J. & Abecasis, G. R. Fast and accurate genotype imputation in genome-wide association studies through pre-phasing. *Nat. Genet.* **44,** 955–959 (2012).
7. Fuchsberger, C., Abecasis, G. R. & Hinds, D. A. minimac2: faster genotype imputation. *Bioinformatics* **31,** 782–784 (2015).
8. Consortium, T. I. H. 3. Integrating common and rare genetic variation in diverse human populations. *Nature* **467,** 52–58 (2010).

Methods S2 – Creating the school type variable

To create the school type variable for the present study, we used TEDS data in combination with data from the National Pupil Database (NPD; https://www.gov.uk/guidance/national-pupil-database-apply-for-a-data-extract).

TEDS data

When the individuals in our sample were 18, they received a questionnaire that included a series of questions asking what type of school they attended during their GCSEs. Respondents were asked to indicate either 'Yes' or 'No' for different school types, including: home-school, comprehensive school, grammar school, independent (private) school, special school, sixth-form or further education college, faith school, academy and single-sex school. Respondents could select 'Yes' to more than one school type.

We classed all respondents who said they went to either a comprehensive or an academy school as 'State non-selective'. Because individuals were able to select more than one school type, we excluded those who also said they went to a grammar school (n = 22), independent school (n = 26) or special school (n = 17). We did not include 'sixth-form' or 'further education college' within the state non-selective school type as we did not have any information about their selection criteria. After exclusions, the total number of individuals attending a state non-selective school was 4,780.

To create the 'Grammar' group, we classed all respondents who said they attended a grammar school as 'Grammar'. Again, we excluded those who indicated that they also went to a private school (n = 24), comprehensive school (n = 22) or special school (n = 3). After exclusions, the total number of individuals in this group was 372. We classed all respondents who said they attended a private school as 'Private'. We excluded those who indicated that they also went to a comprehensive school (n = 26), grammar school (n = 24) or special school (n = 8). After exclusions, the total number of individuals in this group was 513. We could not class individuals who indicated that they went to a faith or single sex school only into one of the three school types, as these schools can be state non-selective, grammar or private schools.

National Pupil Database data

In order to increase sample sizes, we also accessed school type information through the National Pupil Database (NPD). NPD is a pupil-level database which matches pupil and school characteristic data to pupil level attainment in England. Within the TEDS sample, 13,392 individuals gave consent for us to access their NPD records, of which 12,717 individuals were successfully matched. Approximately 700 individuals who had given consent lived outside of the England (for example Wales or Scotland), and therefore individuals could not be matched. In addition to pupil-level data on attainment, NPD also includes information on what type of school an individual attended during their GCSEs which is limited in description to one school type (for a list of school types in NPD and corresponding sample sizes in our data, please see Table SM1). Students coded in NPD as attending: 'community', 'voluntary aided', 'voluntary controlled', 'foundation', 'city technology college', 'non-maintained', 'academy sponsor-led', 'academy-converter' or 'free schools' were classed as 'State non-selective' (n = 10,446). Because NPD does not include a separate category for grammar schools, we identified grammar schools using the Department for Education database 'EduBase' which we could link to NPD data through unique school reference numbers (URNs). This identified 314 students attending grammar schools within our NPD records. Therefore, after excluding these individuals, there were 10,132 individuals attending 'State non-selective' schools in NPD and 314 individuals attending grammar schools. Students coded as attending 'other independent' schools in NPD we classed as 'Private' (n = 998).

Table SM1 - Type of establishment code taken from EduBase and sample sizes in full and selected samples

	Full sample		Selected sample	
Value label	Frequency	Percent	Frequency	Percent
Community	4031	35.0	1630	35.8
Voluntary aided	1459	12.7	551	12.1
Voluntary controlled	355	3.1	156	3.4
Foundation	2469	21.5	983	21.6
City Technology College	9	0.1	1	0.0
Community special	20	0.2	0	0.0
Other independent special	10	0.1	0	0.0
Other independent	998	8.7	386	8.5
Foundation special	2	0.0	0	0.0
Pupil referral unit	24	0.2	0	0.0
Further Education	4	0.0	0.0	0.0
Academy, sponsor-led	462	4.0	177	3.9
Academy, converter	1660	14.4	664	14.6
Free School	1	0.0	1	0.0
Total	11504	100.0	4549	100.0

Note: *Selected sample = sample who have educational achievement at 16 (GCSE), genotype data and school type data*

TEDS and NPD accuracy

There were 4186 individuals who had both TEDS data and NPD data. From this, we checked the accuracy of our groupings using descriptive crosstabs (see Table SM2). This shows the agreement between TEDS and NPD school type data. It revealed high accuracy for both the state non-selective and the private school groups. There were 75 individuals who had stated that they attended a grammar school in the TEDS data, but who actually attended a state non-selective school, as indicated by NPD. This is likely due to grammar schools converting to state non-selective schools, but keeping the title ‘grammar’ within their school name. We decided prioritise the NPD data in these cases.

Table SM2 - Cross tabulation comparison of school type data for TEDS and National Pupil Database

		TEDS: Non-selective	TEDS: Grammar	TEDS: Private	NPD accuracy
NPD	Non-selective	3473	75	8	97.67%
NPD	Grammar	2	231	1	98.71%
NPD	Private	3	3	390	98.48%
TEDS accuracy		99.86%	74.76%	97.74%	

Note: *Squares in dark grey represent individuals who were classed in NPD and TEDS as attending the same school type. Squares in light grey represent those whose school type was different in TEDS and NPD*

School type totals

After combining TEDS and NPD school type data and prioritising NPD data with relation to grammar schools, there were a total of 12,923 individuals for whom we had school type data available. 11,434 attending non-selective state schools, 377 attending grammar schools and 1112 attending private schools. The proportion of students attending the three school types in the current study is representative of UK statistics: for example grammar school UK intake = ~4%[1], our sample = 2.9%; private school UK intake = ~7%[2], our sample = 8.6%.

Of this final number 4,814 also had GCSE data and genotype information, with 4,263 attending non-selective schools, 143 attending grammar school and 408 attending private schools. 2533 people also had data for the selection factors: family SES, prior ability and prior achievement.

State non-selective schools and local education authorities

Local education authorities (LEAs) are the local councils in England and Wales that are responsible for education within their jurisdiction. They can be non-selective (contains no grammar schools), partially selective (contains one or more grammar school) or wholly selective (over 25% of pupils in that LEA attends a grammar school). Previous research suggests that those attending non-selective schools in wholly selective areas perform worse than those in non-selective areas, so we further split our ‘State non-selective’ school type into three subcategories to test this.

Non-selective, partially selective and wholly selective local education authorities (LEAs) were identified from The Education (Grammar School Ballots) Regulations 1998[3], which includes 10 'wholly-selective' LEAs and a further 26 partially selective LEAs. We matched this information to our own data through school LEA.

There were 331 students attending a non-selective school in a wholly selective area, 905 students attending a non-selective school in a partially selective area, and 3,027 students attending a non-selective school in a non-selective area. Numbers for grammar (n = 143) and private (n = 408) schools remained the same.

References:

1 Bolton, P. (2017) *Grammar school statistics*. Briefing Paper 1398. House of Commons Library. Accessed at: http://researchbriefings.parliament.uk/ResearchBriefing/Summary/SN01398#fullreport

2 *Independent Schools Council Census and Annual Report (*2017). Accessed at: https://www.isc.co.uk/media/4069/isc-census-2017-final.pdf

3 The Education (Grammar School Ballots) Regulations 1998: http://www.legislation.gov.uk/uksi/1998/2876/schedule/1/made

Methods S3 – Hierarchical linear regression to calculate adjusted means for school type

To test the effect of school type on GCSE once selection factors (SES, prior achievement and prior ability) had been controlled for, we conducted hierarchical linear regression. In the first step, we entered the selection factors, which were first standardized so that the mean of these variables was 0, and in the second step of the model we entered school type. Because school type is a nominal variable with three categories (non-selective state school, grammar school and private school) without intrinsic ordering, we created two dummy coded variables to represent the three categories. This is a common way of entering nominal variables into multiple linear regression in order to capture all of the categories. Dummy coding requires one of the categories to be the reference category, in which the other categories are compared with; in this analysis we chose to use state non-selective schools as the reference category to look at the effects of selective schools on GCSE performance (see Supplementary Methods S3 for further information).

Conducting hierarchical linear regression enables us to observe the R^2 change between the two steps in the model, indicating the amount of variance in mean GCSE score explained by school type once selection factors have been controlled for. In addition, it also allows us to test whether mean GCSE score differs between school types whilst keeping the selection factors constant. For example, in the case of grammar schools, the mean would be calculated using the equation below:

$$\hat{Y} = \beta_0 + \beta_1 X_1 + \beta_2 X_2 + \beta_3 X_3 + \beta_4 X_4$$

Where $\hat{Y}$ is the mean GCSE for grammar schools, β_0 is the intercept in the second step of the model which, in this case, is the expected mean GCSE of state non-selective schools when all other independent variables are 0 (which have been standardized so that 0 represents their mean), X_1, X_2, X_3 and X_4 are the independent variables: school type, SES, prior ability and prior achievement and β_1, β_2, β_3 and β_4 are the beta coefficients associated with the change in dependent variable when school type goes from state non-selective school to grammar school, whilst keeping the other independent variables constant. We observed the t statistic and its associated significance in order to see whether the mean GCSE differed between groups, once accounting for selection factors.

Table S1 – Analysis of variance (ANOVA) and planned contrasts for *EduYears* GPS between students of three school types: state non-selective, grammar and private schools

				ANOVA		Contrasts			
	n	Mean (*SD*)	95% CIs	*F*	η^2	Contrasts	*t*	d_{cohen}	95% CI
Non-selective (N)	4263	-.043 (1.000)	-.073 to -.014			G vs N	4.869***	.413	.246-.579
Grammar (G)	143	.368 (0.989)	.204 to .531	35.800***	.014	G vs P	.436	N/A	N/A
Private (P)	408	.325 (0.954)	.233 to .418			P vs N	7.170***	.372	.270-.473

Note: *n* = number of participants in each group; *SD* = standard deviation; 95% CIs = 95% confidence intervals around the mean; *F* = test of overall ANOVA model; η^2 = eta squared variance explained; N = non-selective state school students; G = grammar school students; P = private school students; d_{cohen} = adjusted cohen's d statistic; CI = confidence intervals; *** = $p < .001$

Table S2 – Analysis of variance (ANOVA) and planned contrasts for *EduYears* GPS between students of five school types: non-selective schools in wholly selective areas, non-selective schools in partially selective areas, non-selective schools in non-selective areas, grammar schools and private schools

				ANOVA		Contrasts			
	n	Mean (*SD*)	95% CIs	*F*	η^2	Comparison	*t*	d_{cohen}	95% *CI*
**State non-selective schools**						WS vs PS	1.362	*N/A*	*N/A*
Wholly selective area (WS)	331	.002 (1.010)	-.107 to .112			WS vs NS	0.673	*N/A*	*N/A*
Partially selective area (PS)	905	-.084 (1.001)	-.150 to -.019	18.503***	.015	WS vs G	-3.675***	.369	(.248-.489)
Not selective area (NS)	3027	-.036 (.994)	-.072 to -.001			WS vs P	-4.398***	.330	(.184-.476)
**Selective schools**						PS vs NS	-1.280	*N/A*	*N/A*
Grammar (G)	143	.368 (.989)	.204 to .531			PS vs G	-5.058***	.452	(.275-.630)
Private (P)	408	.325 (.954)	.233 to .418			PS vs P	-6.923***	.415	(.297-.532)
						NS vs G	-4.752***	.407	(.239-.575)
						NS vs P	-6.908***	.365	(.261-.469)

Note: *n* = number of participants in each group; *SD* = standard deviation; 95% CIs = 95% confidence intervals around the mean; *F* = test of overall ANOVA model; η^2 = eta squared variance explained; WS = non-selective school in wholly selective area; PS = non-selective school in partially selective area; NS = non-selective school in non-selective area; N = non-selective state school students; G = grammar school students; P = private school students; d_{cohen} = adjusted Cohen's d statistic; *CI* = confidence intervals.* = *p* <.05; ** = *p* < .01; *** = *p* < .001.

Table S3 – Correlation matrix

	EduYears GPS	GCSE	Prior ability	Prior achievement	SES	School type
EduYears GPS	1					
GCSE	.277***	1				
Prior ability	.146***	.524***	1			
Prior achievement	.229***	.805***	.512***	1		
SES	.256***	.493***	.318***	.380***	1	
School type	.121***[a]	.300***[a]	.175***[a]	213***[a]	0.306***[a]	1

***Note**: GPS = genome-wide polygenic score; GCSE = General Certificate of Secondary Education; prior ability = general cognitive ability based on verbal and non-verbal tests at age 11; prior achievement = achievement scores in English and maths at age 11; SES = socio-economic status. [a] = School type correlations obtained through regression using dummy-coded variables; *** = p<.001.*

Table S4 – Hierarchical regression analysis of EduYears GPS, controlling for selection factors for students of three school types: state non-selective, grammar and private schools

	Step 1				Step 2			
	B (95% CIs)	Std. Error	Beta	t	B (95% CIs)	Std. Error	Beta	t
**Constant**	.021 (-.015-.058)	.019		1.141	.007 (-.032-.045)	.020		.346
**Selection factors**								
SES	.172 (.132-.213)	.020	.176	8.431***	.165 (.124-.206)	.0210	.168	7.941***
Prior ability	.011 (-.032-.054)	.022	.011	.500	.009 (-.034-.052)	.022	.009	.400
Prior achievement	.152 (.107-.196)	.023	.155	6.696***	.146 (.101-.191)	.023	.149	6.375***
**School types**								
Non-selective vs Grammar					.202 (-.012-.415)	.109	.036	1.853
Non-selective vs Private					.145 (-0.18-.308)	.083	.034	1.739
	**Model statistics - Step 1**				**Model statistics - Step 2**			
R^2 (Std. Error)	.079 (.078)				.081			
R^2 change	.079				.002			
F Change	72.294***				3.007			

Note: SES = Socioeconomic status; CIs = confidence intervals; * p < 0.05; ** p < 0.01; *** p < 0.001. School type was dummy-coded into two variables with state non-selective schools as the reference category. Constant = mean of state non-selective schools when all other variables held constant; Model step 1: selection factors (SES, prior ability and prior achievement) were entered into the model; Model step 2: selection factors and school type were entered into the model together.

Table S5 – Hierarchical regression analysis of EduYears GPS, controlling for selection factors for students of five school types: non-selective schools in wholly selective areas, non-selective schools in partially selective areas, non-selective schools in non-selective areas, grammar schools and private schools

	Step 1				Step 2			
	B (95% CIs)	Std. Error	Beta	t	B (95% CIs)	Std. Error	Beta	t
**Constant**	.021 (-.015-.058)	.019		1.141	.182 (.043-.321)	.071		2.563*
**Selection factors**								
SES	.172 (.132-.213)	.020	.176	8.431**	.168 (.127-.208)	.021	.171	8.069***
Prior ability	.011 (-.032-.213)	.022	.011	0.5	.007 (-.036-.050)	.022	.007	.310
Prior achievement	.152 (.107-.196)	.023	.155	6.696***	.148 (.103-.192)	.023	.151	6.451***
**School types**								
NS_WS vs NS_NS					-.182 (-.328--.036)	.075	-.089	-2.442*
NS_WS vs NS_PS					-.212 (-.378--.051)	.082	-.087	-2.579*
NS_WS vs G					.025 (-.228-.277)	.129	.004	.192
NS_WS vs P					-.032 (-.244-.179)	.108	-.008	-.300
	**Model statistics for step 1**				**Model statistics for step 2**			
R^2 (Std. Error)	.079 (0.941)				0.084 (0.939)			
R^2 change	.079				.005			
F Change	72.294***				3.252*			

Note: SES = Socioeconomic status; CIs = confidence intervals; NS_WS = State non-selective school in wholly selective area; NS_PS = State non-selective school in partially selective area; NS_NS = State non-selective school in non-selective area; G = Grammar school; P = Private school; * $p < 0.05$; ** $p < 0.01$; *** $p < 0.001$. School type was dummy-coded into two variables with state non-selective schools as the reference category. Constant = mean of state non-selective schools when all other variables held constant. Model step 1: selection factors (SES, prior ability and prior achievement) were entered into the model; Model step 2: selection factors and school type were entered into the model together.

Table S6 – Regression analysis of predictors of mean GCSE for three school types: state non-selective, grammar and private schools

	School type on GCSE (unadjusted)				School type on GCSE controlling for *EduYears*				School type on GCSE controlling for SES			
	B (95% CIs)	Std. Error	Beta	t	B (95% CIs)	Std. Error	Beta	t	B (95% CIs)	Std. Error	Beta	t
**Constant**	8.889 (8.842-8.936)	0.024		370.98***	8.893 (8.848-8.939)	0.023		383.832***	8.927 (8.885-8.969)	0.021		415.360***
**School types**												
N vs G	1.226 (0.969-1.483)	0.131	0.180	9.361***	1.086 (0.837-1.335)	0.127	0.159	8.547***	0.894 (0.663-1.125)	0.118	0.131	7.581***
N vs P	1.066 (0.871-1.260	0.099	0.206	10.741***	0.951 (0.762-1.140)	0.096	0.184	9.867***	0.574 (0.396-0.752)	0.091	0.111	6.313***
**EduYears GPS**					0.303 (0.258-0.347)	0.023	0.248	13.311***				
**Selection factors**												
SES									0.530 (0.488-0.571)	0.021	0.443	25.125***
Prior ability												
Prior achievement												
**Model statistics**												
R^2 (Std. Error)	0.071 (1.152)				0.132 (0.113)				0.257 (1.030)			
R^2 change	0.071				0.056				0.027			
F Change	97.243***				81.099***				45.665***			

Note: Table continues on the next page

	School type on GCSE controlling for ability				School type on GCSE controlling for achievement				School type on GCSE controlling for everything			
	B (95% CIs)	Std. Error	Beta	t	B (95% CIs)	Std. Error	Beta	t	B (95% CIs)	Std. Error	Beta	t
**Constant**	8.919 (8.879-8.960)	0.021		432.032***	8.947 (8.918-8.976)	0.015		604.673***	8.960 (8.933-8.987)	0.014		646.634***
**School types**												
N vs G	0.875 (0.653-1.097)	0.113	0.128	7.721***	0.259 (0.98-0.420)	0.082	0.038	3.159**	0.180 (0.30-0.331)	0.077	0.026	2.346*
N vs P	0.728 (0.559-0.897)	0.086	0.141	8.445***	0.571 (0.450-0.692)	0.062	0.110	9.266***	0.362 (0.246-0.477)	0.059	0.070	6.155***
***EduYears* GPS**									0.079 (0.052-0.107)	0.014	0.065	5.649***
**Selection factors**												
SES									0.194 (0.164-0.223)	0.015	0.162	13.038***
Prior ability	0.595 (0.556-0.634)	0.020	0.498	29.787***					0.150 (0.120-0.181)	0.016	0.126	47.162***
Prior achievement					0.928 (0.900-0.957)	0.014	0.777	64.265***	0.767 (0.735-0.799)	0.016	0.642	9.639***
**Model statistics**												
R^2 (Std. Error)	0.312 (0.991)				0.647 (0.710)				0.692 (0.664)			
R^2 change	0.034				0.013				0.005			
F Change	61.939***				46.191***				20.726***			

Note: SES = Socioeconomic status; CIs = confidence intervals; School type was dummy-coded into two variables with state non-selective schools as the reference category. **Constant** = mean of state non-selective schools when all other variables held constant. N = non-selective state school; G = grammar school; P = private school. **Model statistics**: R^2 = total variance explained by all of the predictors in the model; R^2 change = additional variance added by school type over and above other predictors; * $p < 0.05$; ** $p < 0.01$; *** $p < 0.001$.

Table S7 – Regression analysis of predictors of mean GCSE for three school types: non-selective schools in wholly selective areas, non-selective schools in partially selective areas, non-selective schools in non-selective areas, grammar schools and private schools

	School type on GCSE (unadjusted)				School type on GCSE controlling for EduYears				School type on GCSE controlling for SES			
	B (95% CIs)	Std. Error	Beta	t	B (95% CIs)	Std. Error	Beta	t	B (95% CIs)	Std. Error	Beta	t
**Constant**	8.730 (8.560-8.899)	0.087		100.887***	8.698 (8.534-8.862)	0.084		103.961***	8.874 (8.722-9.027)	0.078		114.233***
**School types**												
NS_WS vs NS_NS	0.178 (0.357--0.089)	0.091	0.072	1.958	0.215 (0.042-0.388)	0.088	0.086	2.439*	0.062 (-0.099-0.222)	0.082	0.025	0.755
NS_WS vs NS_PS	0.154 (0.351--0.044)	0.100	0.052	1.531	0.203 (0.012-0.393)	0.097	0.068	2.084*	0.043-0.0134-0.219)	0.090	0.014	0.476
NS_WS vs G	1.3851.689-0.170)	0.155	0.203	8.932***	1.281 (0.987-1.575)	0.150	0.188	8.534***	0.9470.673-1.222)	0.140	0.139	6.769***
NS_WS vs P	1.225 (1.479-0.198)	0.129	0.237	9.466***	1.146 (0.900-1.391)	0.125	0.221	9.148***	0.628 (0.396-0.860)	0.118	0.121	5.305***
**EduYears GPS**					0.305 (0.260-0.349)	0.023	0.250	13.391				
**Selection factors**												
SES									0.529	0.021	0.443	25.035***
Prior ability												
Prior achievement												
**Model statistics**												
R^2 (Std. Error)	0.073 (1.151)				0.134 (1.113)				0.257 (1.031)			
R^2 change	0.073				0.058				0.027			
F Change	49.619***				42.106***				22.973***			

Note: Table continues on the next page

	School type on GCSE controlling for prior ability				School type on GCSE controlling for achievement				School type on GCSE controlling for everything			
	B (95% CIs)	Std. Error	Beta	t	B (95% CIs)	Std. Error	Beta	t	B (95% CIs)	Std. Error	Beta	t
**Constant**	8.738 (8.592-8.883)	.074		117.421***	8.926 (8.821-9.030)	.053		166.980***	8.938 (8.560-8.899)	0.050		178.166***
**School types**												
NS_WS vs NS_NS	0.208 (0.055-0.362)	0.078	0.084	2.660**	0.016	0.056	0.006	0.284	0.019 (-0.085-0.122)	0.053	0.007	0.353
NS_WS vs NS_PS	0.159 (-0.010-0.329)	0.086	0.053	1.843	0.048	0.062	0.016	0.778	0.040 (-0.074-0.154)	0.058	0.013	0.690
NS_WS vs G	1.056 (0.794-1.319)	0.134	0.155	7.892***	0.281	0.097	0.041	2.893**	0.202 (0.026-0.024)	0.091	0.030	2.223*
NS_WS vs P	0.909 (0.690-1.128)	0.112	0.176	8.127***	0.593	0.080	0.115	7.373***	0.384 (0.234-0.534)	.076	0.074	5.029***
**EduYears GPS**									0.080 (0.052-0.107)	0.014	0.065	5.671***
**Selection factors**												
SES									0.193 (0.164-0.222)	0.015	0.162	12.984***
Prior ability	0.596	0.020	0.499	29.856***					0.150 (0.120-0.181)	0.016	0.126	9.621***
Prior achievement					0.928	0.014		64.181***	0.767 (0.735-0.799)	0.016	0.642	47.073***
**Model statistics**												
R^2 (Std. Error)	0.315 (0.990)				0.647 (0.710)				0.692 (0.664)			
R^2 change	0.036				0.013				0.005			
F Change	32.889***				23.331***				10.510***			

Note: SES = Socioeconomic status; CIs = confidence intervals; School type was dummy-coded into two variables with state non-selective schools as the reference category. **Constant** = mean of state non-selective schools in wholly selective area when all other variables held constant; NS_WS = State non-selective school in wholly selective area; NS_PS = State non-selective school in partially selective area; NS_NS = State non-selective school in non-selective area; G = Grammar school; P = Private school. **Model statistics**: R^2 = total variance explained by all of the predictors in the model; R^2 change = additional variance added by school type over and above other predictors; * $p < 0.05$; ** $p < 0.01$; *** $p < 0.001$.

Table S8 - Descriptive statistics

	Whole sample			Non-selective schools			Grammar schools			Private schools		
	N	Mean	SD	N	Mean	SD	N	Mean	SD	N	Mean	SD
EduYears GPS	4814	.04	1.00	4263	.00	.99	143	.41	.99	408	.36	.95
GCSE	4814	8.84	1.26	4263	8.7	1.24	143	10.01	.77	408	9.85	.96
SES	4574	.20	.98	4034	.09	.95	136	.72	.91	404	1.09	.73
Prior ability[1]	2922	.06	.97	2582	.00	.98	96	.61	.69	244	.49	.78
Prior achievement	4298	68.77	15.63	3935	67.82	15.63	123	84.50	6.17	240	76.32	12.34

Note: *N = number of participants; SD = standard deviation; GCSE = General Certificate of Secondary Education; GPS = genome-wide polygenic score; SES = socioeconomic status. [a] = Full sample using one twin in a pair randomly to maintain independence of data; [1] = standardisation was required to form a composite. For those measures that were standardized, standardization was done on the full sample to show comparison to the selected sample and to the different school types. Descriptives were computed with raw data.*

Figure S1 – *EduYears* GPS plotted means (and 95% confidence intervals) for students of five school types: non-selective schools in wholly selective areas, non-selective schools in partially selective areas, non-selective schools in non-selective areas, grammar schools and private schools

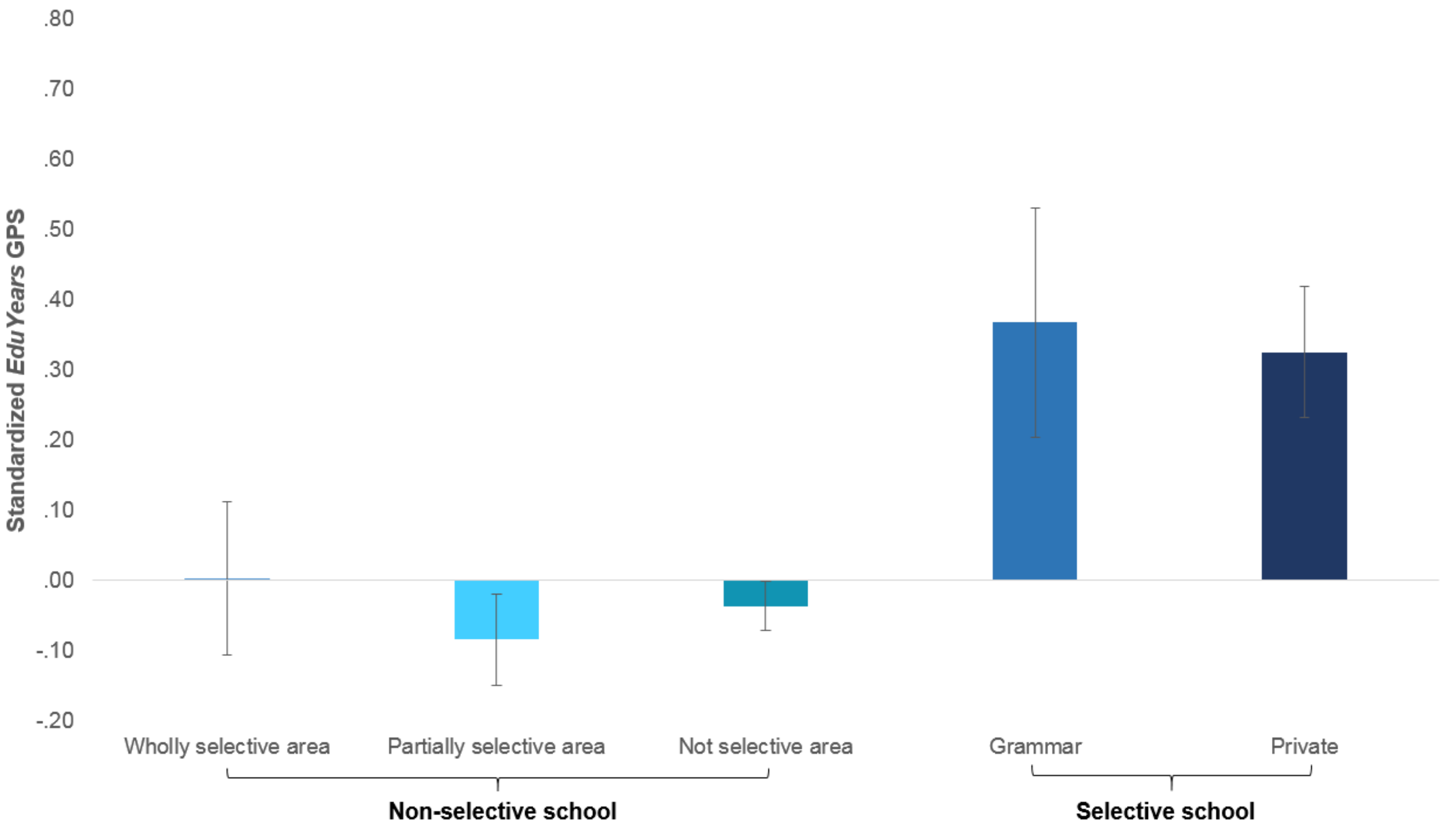

Note: There were no significant mean differences for *EduYears* GPS between state non-selective school students in varying selectively areas (wholly selective vs partially selective t= 1.362, p = .173; wholly selective vs not selective area t= 0.673, p =.501; partially selective vs not selective area t= -1.280, p = .200). There were significant mean differences between all of the state non-selective groups and both grammar and private school students (state wholly selective vs grammar t= -3.675, p <.001; state wholly selective vs private t = -4.398, p <.001; state partially selective vs grammar t= -5.058, p <.001; state partially selective vs private t = -6.923, p <.001; state non selective area vs grammar t= -4.752, p <.001; state not selective area vs private t= -6.908, p <.001).

Figure S2 – *EduYears* GPS plotted means (and 95% confidence intervals) controlling for selection factors between students of 3 school types: non-selective state, grammar and private

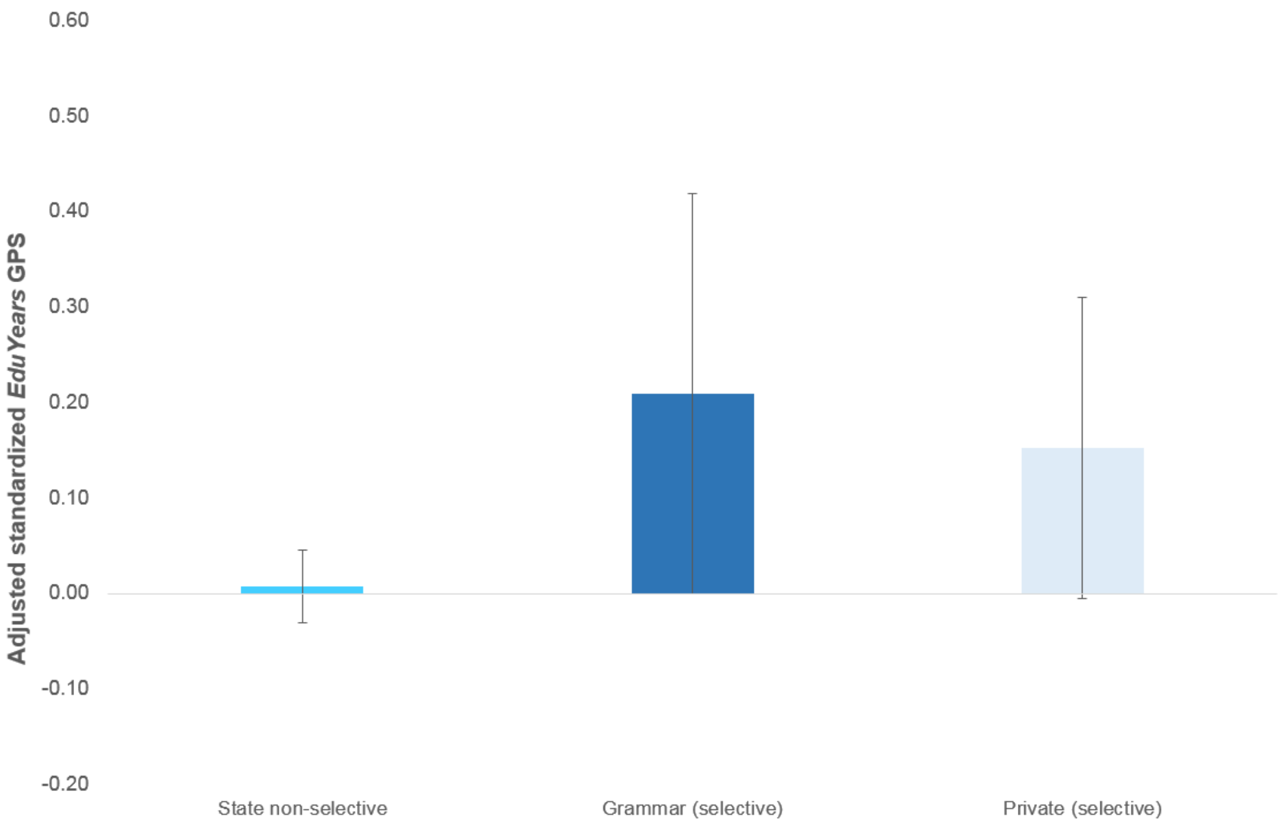

Note: There were no significant *EduYears* GPS mean differences between state non-selective and grammar school students (t = 1.853, p = 0.064) or between state non-selective and private school students (t = 1.739, p = 0.082) or between grammar and private school students (t = .432, p = 0.665). The 95% confidence intervals are larger here than in Figure 1 because the sample sizes were reduced when data for the three selection factors were required (N = 2533).

Figure S3 – *EduYears* GPS plotted means (and standard errors) controlling for selection factors between 5 school types: non-selective schools in wholly selective areas, non-selective schools in partially selective areas, non-selective schools in non-selective areas, grammar schools and private school

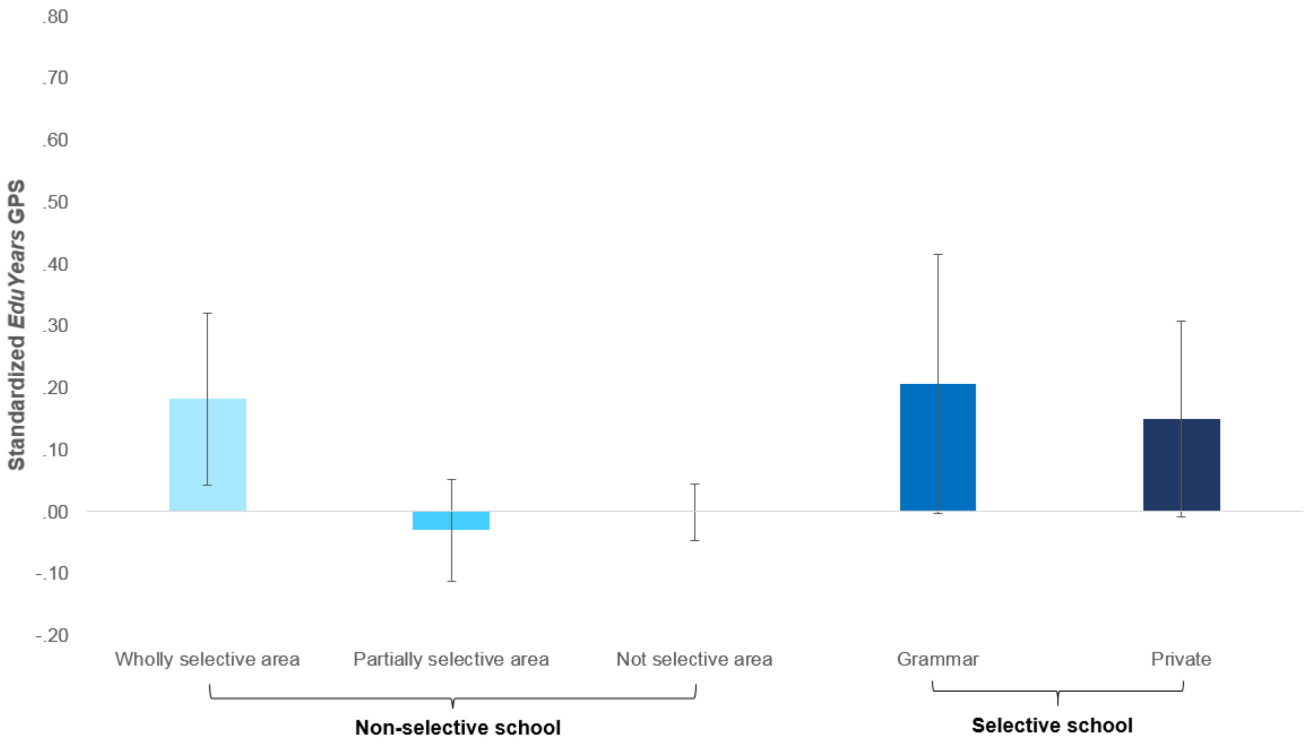

Note: There were small significant differences between students in state non-selective schools in wholly-selective vs partially selective areas (t= -2.579, p =.010) and students in wholly selective areas vs non-selective area (t= -2.442, p = .015), controlling for selection factors. The 95% confidence intervals are larger here than in Figure S1 because the sample sizes were reduced when data for the three selection factors were required (N = 2533).

Figure S4 – The plotted means (and 95% confidence intervals) for unadjusted GCSE, GCSE controlling for *EduYears* GPS, GCSE controlling for SES, GCSE controlling for prior ability, GCSE controlling for prior achievement and GCSE controlling for all variables between 5 school types: non-selective schools in wholly selective areas, non-selective schools in partially selective areas, non-selective schools in non-selective areas, grammar schools and private school

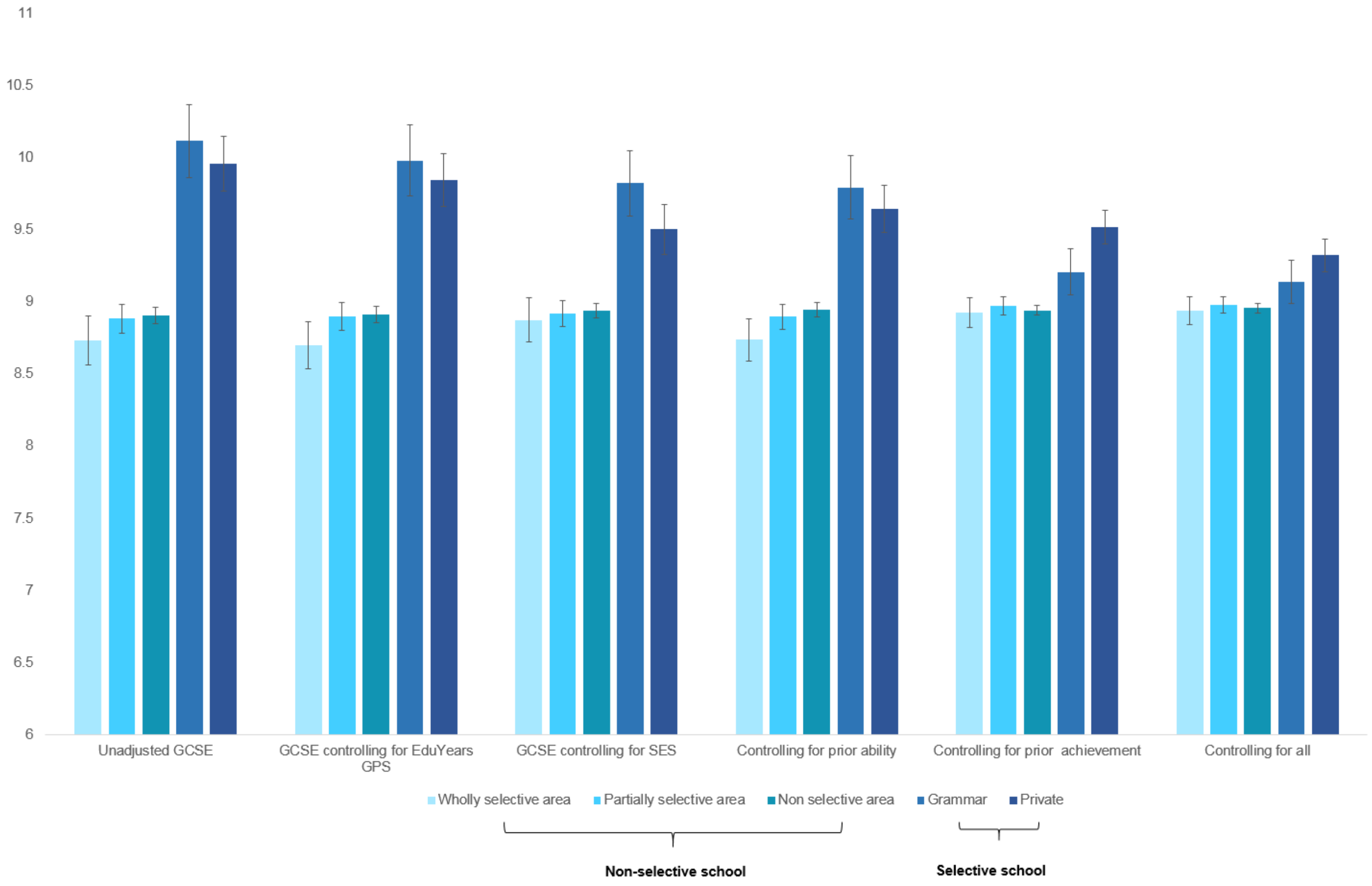

Note: For GCSE controlling for all the variables, there were no differences between non-selective school students in varying selectivity areas. However, there were differences between wholly-selective and both grammar (t = 2.223, p = .026) and private (t = 5.029, p <.001) and between partially selective areas and both grammar (t = 1.997, p = .046) and private (t = 5.348, p <.001) and non-selective and both grammar (t = 2.375, p = .018) and private (t = 6.146, p <.001).

Figure S5 – Mean *EduYears* GPS (and 95% confidence intervals) between state non-selective, grammar and private school for several *EduYears* GPS p-value cut-off

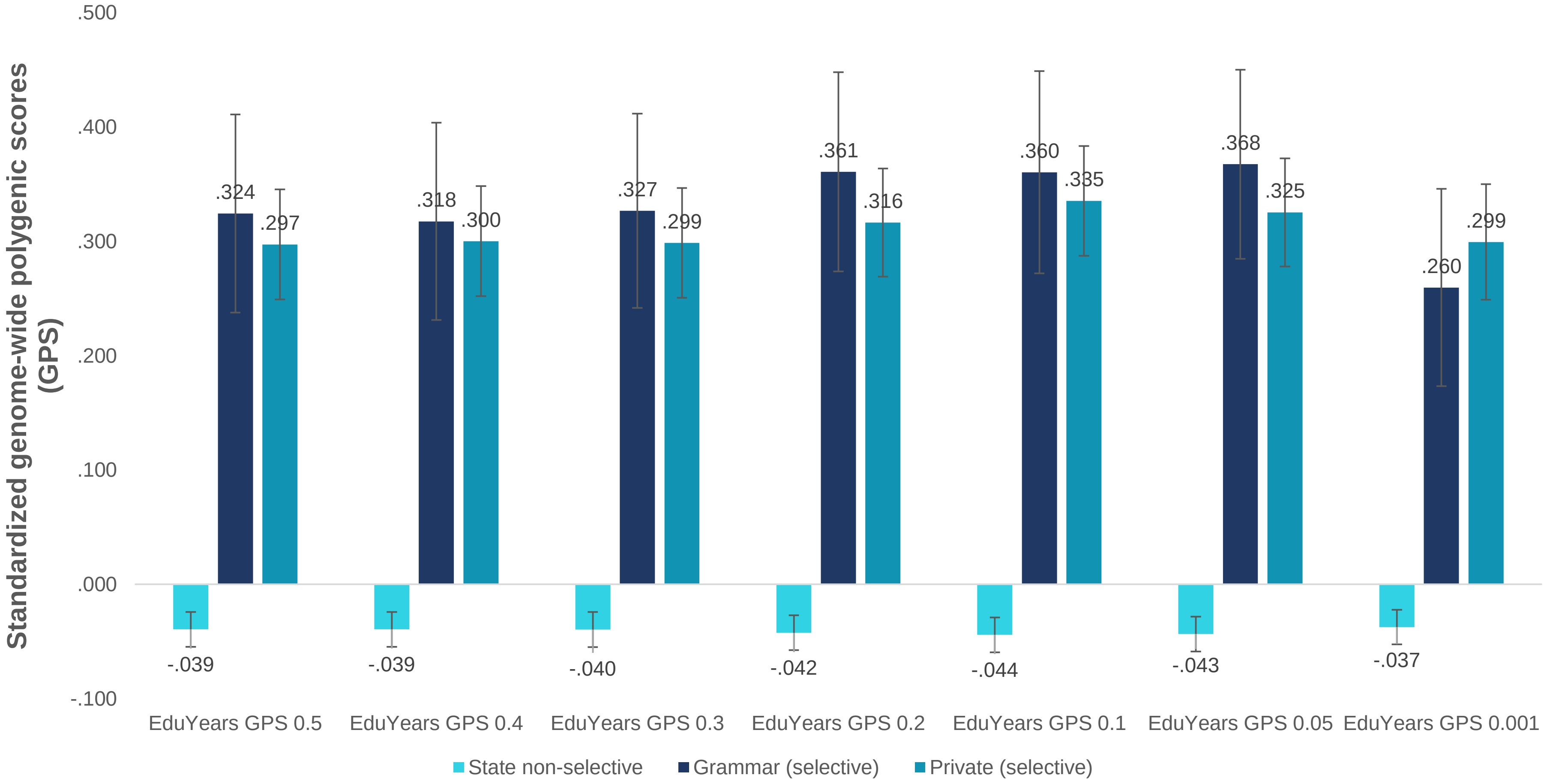

Supplement: Supplementary file 1 — Supplementary Materials [file 41539_2018_19_MOESM1_ESM.pdf]
